# Dielectric Relaxation in the Hybrid Epoxy/MWCNT/MnFe_2_O_4_ Composites

**DOI:** 10.3390/polym12030697

**Published:** 2020-03-21

**Authors:** Darya Meisak, Jan Macutkevic, Artyom Plyushch, Polina Kuzhir, Algirdas Selskis, Juras Banys

**Affiliations:** 1 Vilnius University, Sauletekio Ave. 3, LT-001222 Vilnius, Lithuania; dariameysak@gmail.com (D.M.); artyom.plyushch@gmail.com (A.P.); juras.banys@ff.vu.lt (J.B.); 2 Center for Physical Science and Technology, Sauletekio Ave. 3, LT-001222 Vilnius, Lithuania; algirdas.selskis@ftmc.lt; 3 Institute for Nuclear Problems, Belarusian State University, Minsk 220006, Belarus; 4 Institute of Photonics, University of Eastern Finland, Yliopistokatu 7, FI-80101 Joensuu, Finland; polina.kuzhir@uef.fi

**Keywords:** epoxy, carbon nanotubes, electrical properties, MnFe_2_O_4_, DC conductivity, transport

## Abstract

The electrical properties of epoxy/MWCNT (multi-walled carbon nanotubes)/MnFe_2_O_4_ hybrid composites loaded with MWCNTs (below, 0.09 vol.%, and above, 0.58 vol.%, percolation threshold) and varying concentrations of MnFe_2_O_4_ up to 10 vol.% were studied in a wide frequency range (20 Hz–40 GHz) at different temperatures (20 K–500 K). At low frequencies, the dielectric permittivity and the electrical conductivity of composites with fixed amounts of MWCNT are strongly dependent on MnFe_2_O_4_ content. For MWCNT concentrations above the percolation threshold (i.e., 0.58 vol.%), the electrical conductivity highly decreases with the increase of the MnFe_2_O_4_ fraction. In contrast, for the epoxy/MWCNT just below the onset of electrical conductivity (0.09 vol.% of MWCNTs), there exists an optimal concentration of MnFe_2_O_4_ inclusions (i.e., 0.025 vol.%), leading to a dramatic increase of the electrical conductivity by three orders of magnitude. The electrical transport in composites is mainly governed by electron tunneling at lower temperatures (below 200 K), and it is highly impacted by the matrix conductivity at higher temperatures (above 400 K). The electrical properties were discussed in terms of the Maxwell–Wagner relaxation and distributions of relaxation times. A non-invasive platform based on dielectric relaxation spectroscopy was proposed for enhancing the synergetic effect coursed by using multiple nanoinclusions in polymer composites just below the percolation threshold.

## 1. Introduction

Polymer composites with nanoinclusions are among the most prospective materials for investigations and applications due to the possibility of controlling and improving the macroscopic properties of polymers by the addition of a small amount of nanoparticles [1]. Epoxy resin, due to its attractive mechanical and thermal properties, is a very popular polymer matrix for composite preparation [2,3]. For the electrical properties of composites composed of conducting inclusions and an insulating matrix, the electrical percolation threshold (critical concentration) is the most important parameter [4]. At this concentration, a sharp insulator-conductor transition occurs. The most promising additives for polymer composites are carbon based nanoparticles: carbon nanotubes (CNTs), graphene, and carbon black [4,5,6]. Composites with these inclusions exhibit improved conductive, dielectric, thermal, mechanical properties and low percolation threshold values [6,7,8]. However, composites with other nanoinclusions, for example, ferromagnetic and ferroelectric ones, have also attracted the attention of researchers due to the possibility of producing ferromagnetic or ferroelectric polymer materials, respectively [9,10,11].

Hybrid composites containing two or more nanofillers are very interesting due to their synergy effects, which can significantly improve the electrical, mechanical, and thermal properties of the composites [12]. The synergy effects appear to be due to the proper distributions of different types of nanoparticles and the interactions between them [12]. The increase in electrical conductivity in hybrid percolative composites is followed by a decrease in the percolation threshold. Usually, it is assumed that the synergy effect is observed in hybrid percolative composites if the percolation threshold is lower than that predicted by the excluded volume theory [13]. Hybrid composites filled with nanoparticles of different dimensions have most often been studied, for example, carbon black/carbon nanotubes and others [14]. These composites exhibit substantially improved electrical and mechanical properties. Hybrid composites with carbon and magnetic nanoparticles are very promising for microwave electromagnetic shielding applications as they expose high dielectric and magnetic losses [15].

Manganese ferrite (MnFe_2_O_4_) is a very demanding material due to its excellent electrical, optical, and magnetic performance [16]. MnFe_2_O_4_ nanoparticles are widely used for sensors and biomedical applications [17]. Composites with MnFe_2_O_4_ nanoparticles are also utilized in various practical applications [18,19]. It was determined that the percolation threshold in MnFe_2_O_4_ based composites (depending on the nanoparticle size) can be close to 30 vol.% [20]. Moreover, it has been shown that composites with MnFe_2_O_4_ coated multiwalled carbon nanotubes (MWCNTs) are very appealing for electromagnetic shielding tasks [21], although hybrid composites with MnFe_2_O_4_ and MWCNTs have not been investigated until now. 

The aim of this work was to find the synergy between MnFe_2_O_4_ nanoparticles and MWCNTs, if any, in the electrical properties of epoxy resin composites in a wide frequency range.

## 2. Materials and Methods

Commercially available manganese ferrite (MnFe_2_O_4_) nanopowder with spherical particles of 28 nm size [22] and multi-walled carbon nanotubes (MWCNTs) grown by the chemical vapor deposition method [23] were used as fillers. The average mean outer MWCNT diameter was 20–40 nm, whereas the length was 0.5–200 µm. Commercial epoxy resin Epikote 828 was used as the polymer matrix.

The preparation method of hybrid composites (in our case with two different filler types) does not differ essentially from the dispersion of one filler in a polymer matrix [20,24]. The only difference is the separate dispersion of each inclusion (MWCNT and MnFe_2_O_4_) in ethanol by using ultrasonic devices (bath and probe during 1h each of them). Thereafter, epoxy resin was added to the MWCNT/ethanol suspension, and the resulting mixture was processed with an ultrasonic probe for 1 h. This preparation protocol is associated with the high MWCNT ability to form agglomerates (due to their huge aspect ratio), and therefore they require more intense dispersion by ultrasonic treatment. The final 1 h ultrasonic probe treatment took place after mixing together both the MnFe_2_O_4_/ethanol and MWCNT/epoxy/ethanol obtained suspensions. Then, after complete alcohol evaporation, the resulting mixture was cured by triethylenetramine (TETA) [25] hardener for 24 h at room temperature and 2 h at 100 °C in the oven.

Using the above-mentioned preparation method, two separate series of MnFe_2_O_4_/MWCNT/epoxy resin hybrid composites with a fixed MWCNT-content and various MnFe_2_O_4_ amounts were prepared. The first had a MWCNT content of 0.09 vol.% (just below the percolation state in corresponding monofiller composites [26]), and the following MnFe_2_O_4_ concentrations: 0, 0.025, 0.05, 0.35, 0.65, 5, and 10 vol.%, while the second one had a higher MWCNT content of 0.58 vol.% (above percolation threshold) and 0, 0.025, and 0.58 vol.% of MnFe_2_O_4_. The volume concentrations were evaluated from weight concentrations considering that the density of epoxy resin was 1.16 g/cm^3^, MnFe_2_O_4_ was 5.4 g/cm^3^, and MWCNT was 2 g/cm^3^.

The complex dielectric permittivity in the frequency range from 20 Hz to 1 MHz was measured using an LCR meter HP4284A (Hewlett-Packard, Palo Alto, California). Each measurement was followed first by heating to 500 K using a home-made furnace and then by cooling to 30 K by a closed cycle helium cryostat. The dielectric measurements in the frequency range from 1 MHz to 3 GHz were performed with the coaxial line method using a vector network analyzer Agilent 8714ET (Agilent Technologies, Santa Clara, California). A custom built waveguide spectrometer was used for microwave measurements in the frequency range from 8 to 12 GHz. Thin-rod method was utilized in the waveguide [27].

The structure properties and surface morphology was studied by scanning electron microscopy (SEM) using a Helios NanoLab 650 microscope (Thermofisher Scientific, Hillsboro, USA).

## 3. Results and Discussion

### 3.1. Room-Temperature Properties

**Figure 1:**
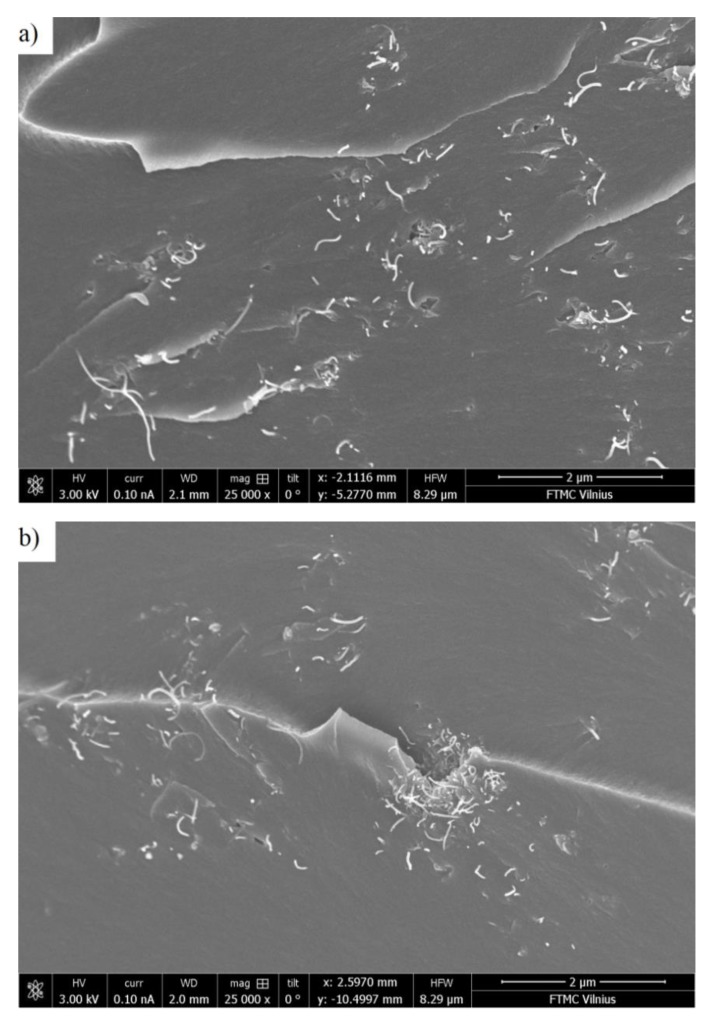

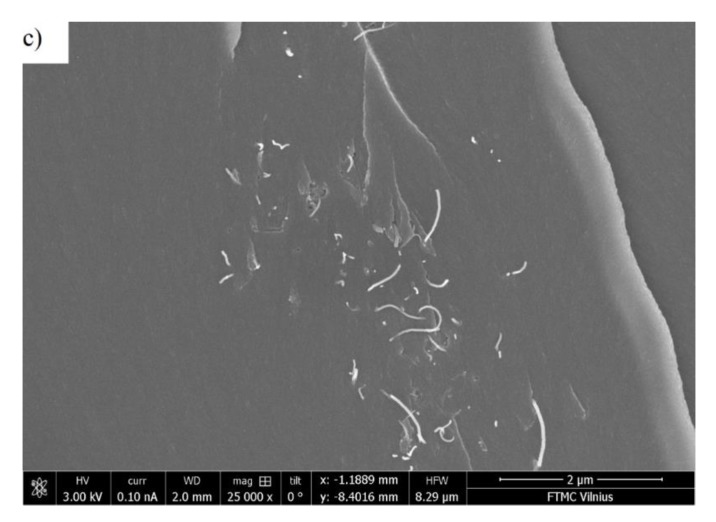
Scanning electron microscopy (SEM) micrographs of the epoxy resin composites with 0.09 vol.% multi-walled carbon nanotubes (MWCNTs) and (**a**) 0.025, (**b**) 0.35, and (**c**) 0.65 vol.% MnFe_2_O_4_ content.

**Figure 2:**
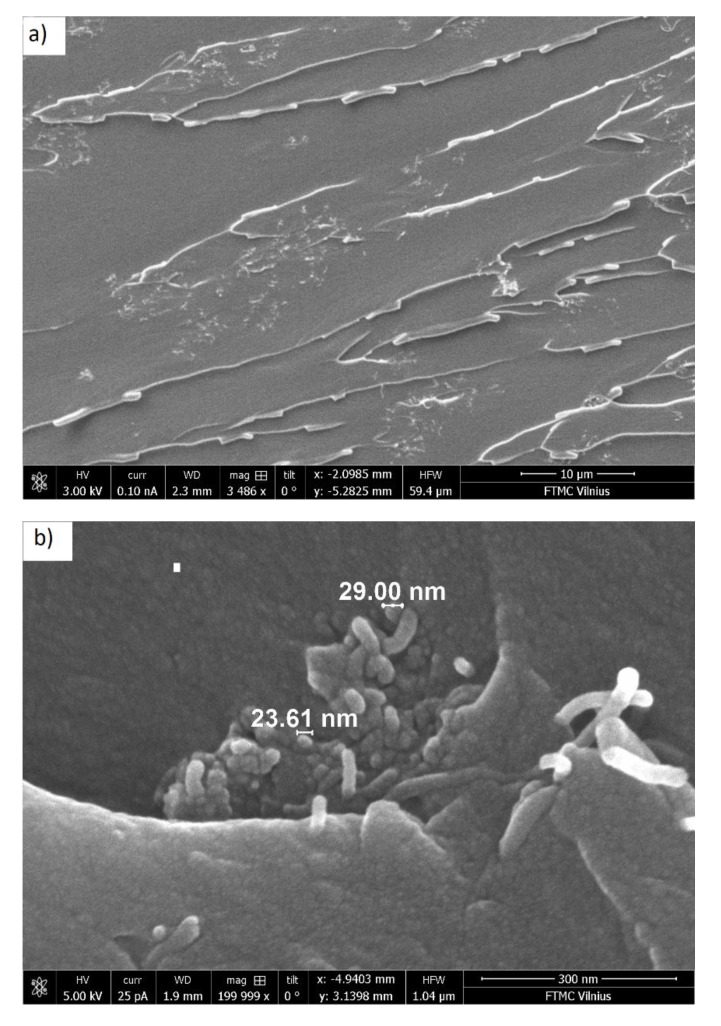
SEM micrographs of the epoxy resin composite with 0.09 vol.% of MWCNTs and 0.025 MnFe_2_O_4_ content at (**a**) low and (**b**) high magnifications.

SEM images of the prepared composites with 0.09 vol.% MWCNT and 0.025, 0.35, and 0.65 vol.% MnFe_2_O_4_ at the same middle magnification (25,000×) are presented at Figure 1. It can be concluded that the best distribution of MWCNTs was observed for composites with the lowest concentration of MnFe_2_O_4_ (0.025 vol.%). With increasing MnFe_2_O_4_ content, MWCNT agglomerates were more pronounced. The SEM-micrograph with low magnification around 3500× (see Figure 2a) proved that MWCNTs could disperse uniformly in the epoxy matrix. Due to the low MnFe_2_O_4_-content and their nanometer particle size, a high magnification (200,000×) was required to detect them (see Figure 2b).

**Figure 3:**
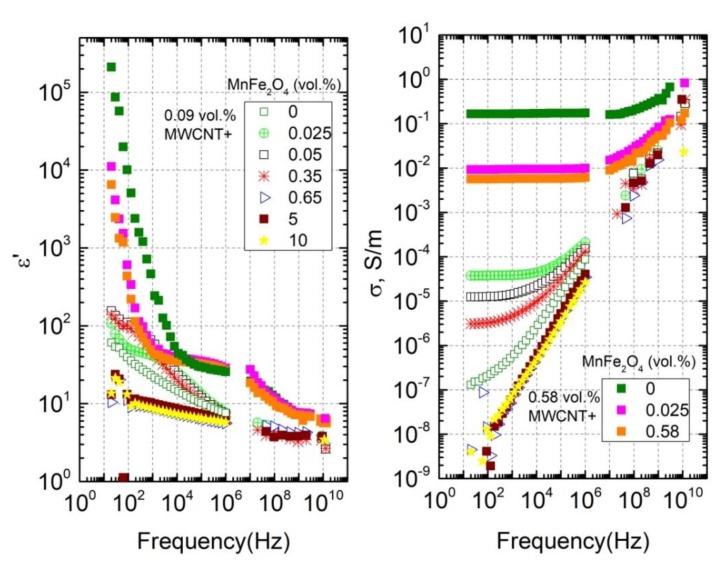
Frequency dependencies of the real part of dielectric permittivity and the electrical conductivity for all composites under study at room temperature.

Frequency dependencies of the real part of dielectric permittivity (ε’) and the electrical conductivity (σ) at room temperature for all composites under study are presented in Figure 3. The electrical conductivity (σ) was calculated from the experimental data of the imaginary part (ε’’) of dielectric permittivity according to $\sigma =2\pi \nu \varepsilon_{0}\varepsilon^{\prime \prime }$, where $\varepsilon_{0}$ is the permittivity of vacuum and ν is the measurement frequency.

At low frequencies (below 1 MHz), the dielectric permittivity and the electrical conductivity of composites are strongly dependent on MnFe_2_O_4_ concentration.

**Figure 4:**
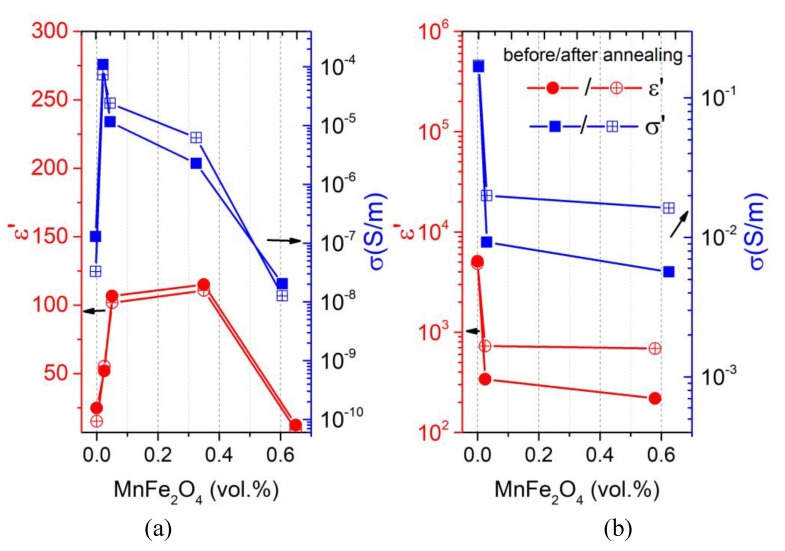
MnFe_2_O_4_-concentration dependencies of the real part of dielectric permittivity and electrical conductivity for composites with (**a**) 0.09 vol.% and (**b**) 0.58 vol.% of MWCNTs at room temperature and 129 Hz before and after annealing at 500 K.

In the case of the first sample series with a MWCNT concentration of 0.09 vol.%, after the addition of a small amount of MnFe_2_O_4_ (0.025 vol.%) to the initially non-conductive composite (open green symbols in the Figure 3), the hybrid composite became conductive and its conductivity value increased by three orders of magnitude. With a further increase in MnFe_2_O_4_ content (0.05 and 0.35 vol.%), the electrical conductivity decreased and at the three highest concentrations (0.65, 5, and 10 vol.%), the samples became fully non-conductive (the $\sigma_{DC}$ conductivity plateau is absent), with σ values even lower than that for composites without MnFe_2_O_4._ This maximum of σ close to the 0.025 vol.% of MnFe_2_O_4_ was visually pronounced on the corresponding concentration dependence at room temperature and 129 Hz, as shown in Figure 4a (the results of ε’ and σ are presented before and after annealing at 500 K).

Thus, in this composite series with a pre-percolation MWCNT content, one can observe a synergy effect between two fillers, which takes place only at certain small amounts of MnFe_2_O_4_. This result can be explained by the analysis of the SEM-micrographs. The best MWCNT distribution was observed for the sample with a minimum MnFe_2_O_4_ concentration. In addition, electrical transport could occur between the MWCNT and MnFe_2_O_4_ clusters, and this mechanism should lead to the rise in the total composite conductivity. However, its contribution to the total conductivity is obviously much smaller than the tunneling conductivity between the MWCNT clusters.

In the case of the second sample series with a high MWCNT concentration of 0.58 vol.%, after the addition of any small (0.025 vol.%) or relatively large (0.58 vol.%) amounts of MnFe_2_O_4_ to the initially conductive composite, the absolute values of electrical conductivity became smaller (see Figure 3 and Figure 4b). Thus, in the case of an initially well-formed percolation network of nanotubes, any amount of non-conductive MnFe_2_O_4_ particles leads to a decrease in electrical conductivity. This means that there is no synergy effect for this sample series. The observed effect can be explained by the worse distribution of MWCNTs after the addition of MnFe_2_O_4_ nanoparticles.

### 3.2. Temperature-Dependent Region

**Figure 5:**
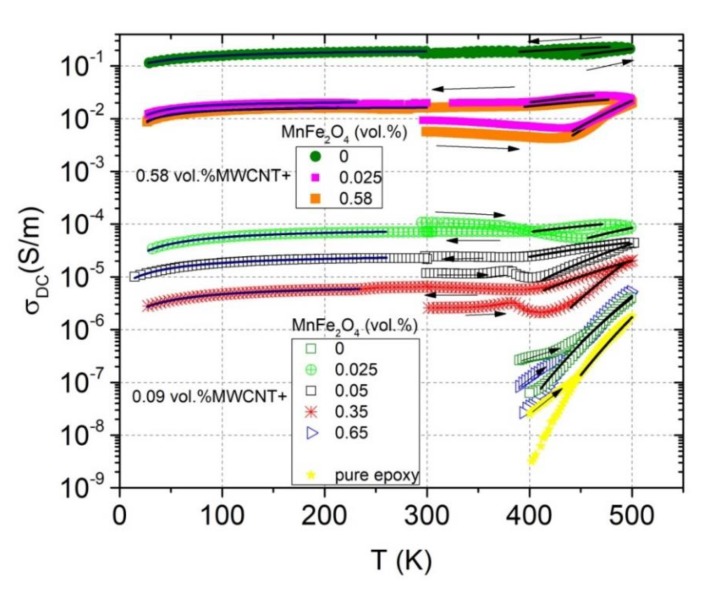
Temperature dependence of the DC conductivity. Solid lines at high and low temperatures correspond to approximations according to Equations (1) and (2), respectively.

The temperature dependencies of DC conductivity in a wide temperature range are presented in Figure 5. For two composites from the first series (with 0.09 vol.% of MWCNT and 0 and 0.65 vol.% of MnFe_2_O_4_), which were initially non-conductive at room temperature, DC conductivity appeared only at high temperatures (above 400 K). This is due to the fact that at high temperatures, the epoxy resin becomes conductive (yellow curve in Figure 5) [28]. A similar DC conductivity behavior was observed for other non-conductive composites at room temperature (5 and 10 vol.% of MnFe_2_O_4_).

For these composites, which are initially conductive at room temperature, the following DC conductivity behavior features were observed. First, during heating from room temperature to 400–450 K (depending on the particular sample), the DC conductivity slightly decreased due to the thermal expansion of epoxy resin and the increase in the distance between the particles [28]. Then, the DC conductivity began to increase up to 500 K (epoxy resin contribution). Cooling from 500 K to room temperature had a similar tendency, the difference was mainly in the absolute value of DC conductivity. Basically, after annealing at 500 K, the DC conductivity at room temperature increased, which indicates that some redistribution of particles in the matrix occurs (large conductive agglomerates broke up into small ones) [28]. However, one composition (with 0.09 vol.% of MWCNTs and 0.025 vol.% of MnFe_2_O_4_) showed the opposite behavior. Due to the smallest MnFe_2_O_4_ and MWCNT concentrations, the distance between the particles was the largest, therefore the percolation network was the most unstable, and its partial destruction was possible after annealing due to the rapid thermal expansion of the polymer matrix. Further cooling from room temperature to 30 K is characterized by a gradual decrease of DC conductivity. The inflections in the electrical conductivity data around 375–400 K can be related to the occurrence of the glass transition [28].

At high temperatures, $\sigma_{DC}$ can be fitted by the Arrhenius law

(1) $$\sigma_{DC}=\sigma_{0}exp\left(\frac{-E_{A}}{k_{B}T}\right)$$

where $\sigma_{0}$ is the preexponential factor; k_B_ is the Boltzmann constant; and $E_{A}$ is the activation energy. Obtained parameters are presented in Table 1. In the composite series with 0.09 vol.% MWCNTs, the activation energy showed the minimum at the MnFe_2_O_4_ concentration of 0.025 vol.%, which corresponded to the maximum of conductivity (see Figure 4a). In the composite series with 0.58 vol.% MWCNTs, the lowest activation energy was observed for the most conductive sample (without MnFe_2_O_4_); for the two other samples, $E_{A}$ had close values. In addition, for both series, the activation energy decreased after annealing. Thus, it can be concluded that the lowest activation energy is typical for the highest electrical conductivity composites as the contribution of the matrix electrical conductivity was insufficient for these composites.

At low temperatures, the DC conductivity fitted well according to the tunneling model [29]:

(2) $$\sigma_{DC}=\sigma_{0}exp\left(\frac{-T_{1}}{T+T_{0}}\right)$$

where $\sigma_{0}$ is the pre-exponential factor; $T_{1}$ represents the energy required for an electron to cross the insulator gap between the conductive particle aggregates; and $T_{0}$ is the temperature above which thermally activated conduction over the barriers begins to occur.

In the tunneling model, parameters $T_{1}$ and $T_{0}$ are determined by $T_{1}=wA\beta_{0}/8\pi k_{B}$ and $T_{0}=2T_{1}/\pi \chi w$ expressions, respectively. Here, $\chi =\sqrt{2mV_{0}}/ћ$ and $\beta_{0}=4V_{0}/ew$; m and e are the electron mass and charge, respectively; $V_{0}$ is the potential barrier amplitude; w is the inter-particles distance (gap width); A is the area of capacitance formed by the junction; ћ is the Dirac constant; and π is the pi number. Obtained parameters are presented in Table 2. The ratio $T_{1}/T_{0}$ is proportional to the gap width w and the potential barrier $V_{0}$ amplitude. Indeed, according to the last column in Table 2, for composites with a 0.09 vol.% of MWCNTs, the highest value of the $T_{1}/T_{0}$ ratio was observed for the lowest concentrations of MnFe_2_O_4_ due to the large potential barrier $V_{0}$ amplitude for particle tunneling. The importance of the potential barrier $V_{0}$ amplitude for the electrical transport mechanism was also clearly observed for hybrid composites with a 0.58 vol.% of MWCNTs, where the ratio $T_{1}/T_{0}$ was the largest for composites with the middle values of the conductivity and distances between nanoparticles. Thus, at low temperatures, the main transport mechanism is electron tunneling through the potential barrier, and MnFe_2_O_4_ is the factor that can tune the potential barrier.

**Figure 6:**
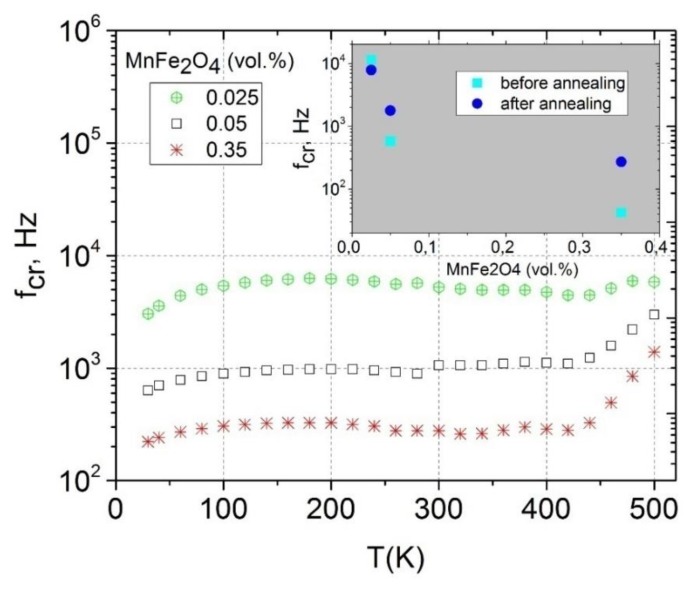
Temperature dependence of critical frequency for composites with 0.09 vol.% of MWCNTs (Insert: critical frequency versus MnFe_2_O_4_ concentration at room temperature before and after annealing at 500 K).

The electrical properties of the composites can also be characterized in terms of the critical frequency. The critical frequency $f_{cr}$ is the frequency at which the DC conductivity plateau passes into the frequency-dependent region. It is possible to calculate $f_{cr}$ from the σ frequency spectra (see Figure 3). The critical frequency for all composites with the MWCNT concentration of 0.58 vol.% was higher than 1 MHz. The results $f_{cr}$ for conductive composites with a MWCNT concentration of 0.09 vol.%. are presented in Figure 6. The temperature dependence (from 500 K to 30 K) of the critical frequency had a basically similar behavior as the temperature dependence of DC conductivity (see Figure 5) for the corresponding samples. The MnFe_2_O_4_ concentration increased, and the critical frequency decreased. The information about the critical frequency before annealing can be obtained from the inset of Figure 6.

The temperature dependence of the critical frequency mainly corresponded to the temperature dependence of DC conductivity according to the relations

(3) $$f_{cr}=\frac{\sigma_{DC}}{\varepsilon_{0}\varepsilon_{s}}$$

(4) $$\sigma_{DC}f_{cr}^{z},$$

where z is an exponent, which characterizes the relation between capacitive and conductive networks in the composite. Above 400 K, both the DC conductivity and critical frequency strongly increased with temperature and the z value was close to 0.5. This value corresponds to the strong variation of conductivity and the weak variation of permittivity on the gaps between clusters [30]. After annealing, the critical frequency also increased together with DC conductivity (Figure 4 and Figure 6 inset).

### 3.3. Relaxation Time Distributions

Experimental data of complex dielectric permittivity can be converted to complex impedance ($Z=Z^{\prime }-iZ^{\prime \prime }$) using the following expressions

(5) $$Z^{\prime }=\frac{\varepsilon^{\prime \prime }}{{\varepsilon^{\prime }}^{2}+{\varepsilon^{\prime \prime }}^{2}}\frac{1}{2\pi f\varepsilon_{0}}$$

(6) $$Z^{\prime \prime }=\frac{\varepsilon^{\prime }}{{\varepsilon^{\prime }}^{2}+{\varepsilon^{\prime \prime }}^{2}}\frac{1}{2\pi f\varepsilon_{0}}$$

**Figure 7:**
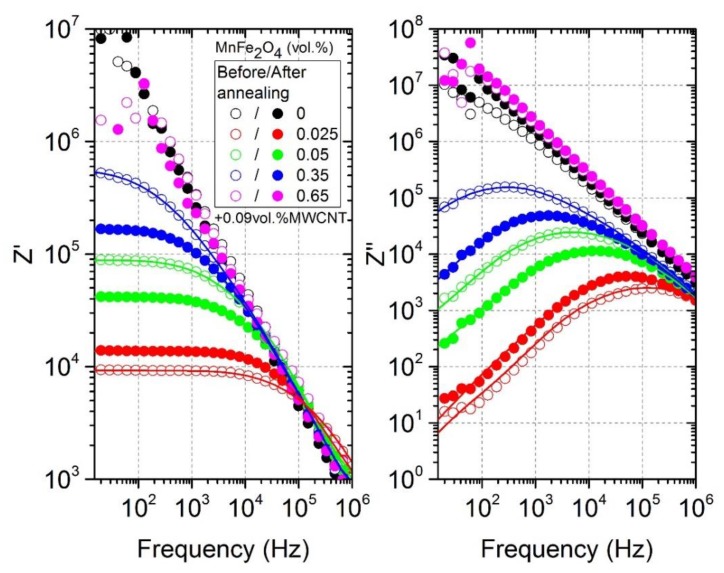
Frequency dependence of the complex impedance before and after annealing at 500 K.

The results are presented in Figure 7. The frequency at which the frequency-independent plateau of $Z^{\prime }$ disappears and the $Z^{\prime \prime }$ has a maximum is close to the critical frequency $f_{cr}$, as discussed earlier. This critical frequency $f_{cr}$ is related to the relaxation time τ by a simple expression

(7) $$2\pi f_{cr}\tau =1$$

However, since the considered composites are a heterogeneous system consisting of particles with a size and shape dispersion, their relaxation time has some distribution f(τ). Since this relaxation is a Maxwell–Wagner one, the relaxation time distribution can be obtained from complex impedance by solving the following integral equation [31]:

(8) $$Z\left(\nu \right)=Z_{\infty }+\Delta Z\int_{-\infty }^{+\infty }\frac{f\left(\tau \right)dlog\tau }{1+i\omega \tau }$$

**Figure 8:**
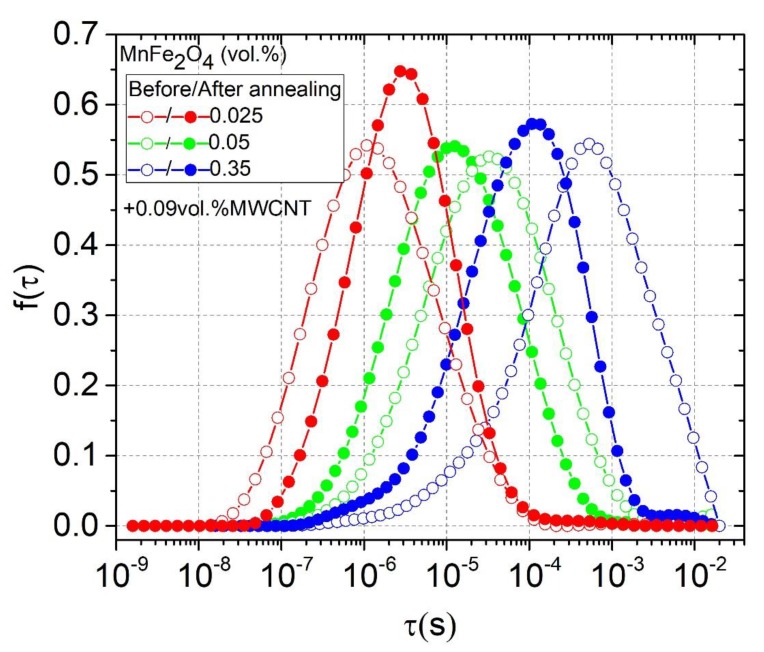
Relaxation time distributions before and after annealing at 500 K.

The obtained distributions of relaxation times before and after annealing are presented in Figure 8. The distributions were calculated only for those samples where the τ value was within the experimental range under consideration. The distributions were symmetrical for all presented samples. The relaxation time of the system was directly related to its conductivity (Equations (4) and (7)), while the distributions of the relaxation times were related to the distribution of nanoparticles inside the polymer matrix in accordance with the data presented in Figure 1 and Figure 2 [32]. Exactly the same pattern can be observed in Figure 8, which completely correlates with the conductivity behavior (see Figure 4a or Figure 5).

## 4. Conclusions

The electrical properties of the epoxy/MWCNT/MnFe_2_O_4_ hybrid composites with two fixed MWCNT amounts (below and above percolation threshold for the case of one-phase composites) and varying MnFe_2_O_4_ concentrations up to 10 vol.% were investigated in the broad frequency (20 Hz–40 GHz) and temperature (20 K–500 K) regions. At low frequencies, the dielectric permittivity and the electrical conductivity of composites are strongly dependent on MnFe_2_O_4_ concentration. Moreover, for composites with MWCNT concentrations just below the percolation threshold, the electrical conductivity had a maximum close to 0.025 vol.% of manganese ferrite, which gave up to 10^3^ larger conductivity than that of the composite without MnFe_2_O_4._ This indicates that a pronounced synergy effect between the two types of particles occurs. In contrast, for composites with MWCNT concentrations above the percolation threshold (for the case of just the polymer comprising MWCNTs), the synergy effect was not observed with any MnFe_2_O_4_ content. The occurrence of synergy effects in the electrical properties of polymer composites is strongly related to the favorable distribution of nanoparticles inside the polymer matrix, which was suggested by the SEM investigations and calculations of the distribution of relaxation times. 

We demonstrate that the dielectric relaxation spectroscopy in polymer based many-phase composites below the percolation threshold could be used as a non-invasive platform for the estimation of the nanoparticle distribution within the bulk of the polymer matrix. The latter is directly related to the synergetic effect from the use of a few different nanoinclusions. To conclude, the analysis of the dielectric relaxation processes in multiphase composites at given fixed concentrations of the conductive functional filler and varying content of an additional one could help to optimize the relative amount of the second filler for achieving synergy.

## Figures and Tables

**Table 1:** Parameters of the Arrhenius law fit.

Sample	Before Annealing		After Annealing	
	$\sigma_{0},\;S/m$	E_A_/k_B_, K	$\sigma_{0},\;S/m$	E_A_/k_B_, K
Pure epoxy	1.3 × 10^4^	11,363	1.6 × 10^5^	12,433
0.09 vol.% MWCNT	2.2 × 10^2^	8892	5.6 × 10^2^	9338
0.09 vol.% MWCNT + 0.025 vol.% MnFe_2_O_4_	9.5 × 10^−3^	2357	6.7 × 10^−4^	898
0.09 vol.% MWCNT + 0.05 vol.% MnFe_2_O_4_	7.5 × 10^−2^	3736	5.7 × 10^−4^	1266
0.09 vol.% MWCNT + 0.35 vol.% MnFe_2_O_4_	2.0 × 10^2^	7998	1.0 × 10^−2^	3103
0.09 vol.% MWCNT + 0.65 vol.% MnFe_2_O_4_	4.1 × 10^4^	11,380	1.7 × 10^3^	9755
0.58 vol.% MWCNT	3.0	1272	0.58 × 10^−1^	446
0.58 vol.% MWCNT + 0.025 vol.% MnFe_2_O_4_	5.0 × 10^2^	5056	1.9 × 10^−1^	897
0.58 vol.% MWCNT +0.58 vol.% MnFe_2_O_4_	1.6 × 10^3^	5623	1.1 × 10^−1^	724

**Table 2:** Tunneling model parameters.

Sample	$\sigma_{0},\;S/m$	$T_{1},\;K$	$T_{0},\;K$	$T_{1}/T_{0}$
0.09 vol.% MWCNT + 0.025 vol.% MnFe_2_O_4_	8.7 × 10^−5^	55.3	26.2	2.1
0.09 vol.% MWCNT + 0.05 vol.% MnFe_2_O_4_	2.8 × 10^−5^	59.5	40.4	1.5
0.09 vol.% MWCNT + 0.35 vol.% MnFe_2_O_4_	7.3 × 10^−6^	59.3	32.9	1.8
0.58 vol.% MWCNT	2.1 × 10^−1^	49.2	47.4	1.0
0.58 vol.% MWCNT + 0.025 vol.% MnFe_2_O_4_	2.2 × 10^−2^	20.7	5.3	3.9
0.58 vol.% MWCNT + 0.58 vol.% MnFe_2_O_4_	1.8 × 10^−2^	27.3	11.6	2.4
